# The Inflammatory Response to Ventricular Assist Devices

**DOI:** 10.3389/fimmu.2018.02651

**Published:** 2018-11-15

**Authors:** Gemma Radley, Ina Laura Pieper, Sabrina Ali, Farah Bhatti, Catherine A. Thornton

**Affiliations:** ^1^Swansea University Medical School, Swansea, United Kingdom; ^2^Calon Cardio-Technology Ltd, Institute of Life Science, Swansea, United Kingdom; ^3^Scandinavian Real Heart AB, Västerås, Sweden; ^4^Department of Cardiology, Morriston Hospital, Abertawe Bro Morgannwg University Health Board, Swansea, United Kingdom

**Keywords:** heart failure, ventricular assist devices, inflammation, cytokines, leukocytes

## Abstract

The therapeutic use of ventricular assist devices (VADs) for end-stage heart failure (HF) patients who are ineligible for transplant has increased steadily in the last decade. In parallel, improvements in VAD design have reduced device size, cost, and device-related complications. These complications include infection and thrombosis which share underpinning contribution from the inflammatory response and remain common risks from VAD implantation. An added and underappreciated difficulty in designing a VAD that supports heart function and aids the repair of damaged myocardium is that different types of HF are accompanied by different inflammatory profiles that can affect the response to the implanted device. Circulating inflammatory markers and changes in leukocyte phenotypes receive much attention as biomarkers for mortality and disease progression. However, they are seldom used to monitor progress during and outcomes from VAD therapy or during the design phase for new devices. Even the partial reversal of heart damage associated with heart failure is a desirable outcome from VAD use. Therefore, improved understanding of the interplay between VADs and the recipient's inflammatory response would potentially increase their uptake, improve patient lives, and fuel research related to other blood-contacting medical devices. Here we provide a review of what is currently known about inflammation in heart failure and how this inflammatory profile is altered in heart failure patients receiving VAD therapy.

## Introduction

Heart failure (HF) is a progressive syndrome which occurs when cardiac structure or function becomes abnormal leading to failure of the heart to deliver oxygen proportionate with the requirements of metabolizing tissues (1). Symptoms of HF are fairly non-discriminating (breathlessness, fatigue, and ankle swelling) which makes early diagnosis challenging, particularly in the elderly and obese (2). HF affects at least 26 million people worldwide (3) with an expected increase in prevalence of 25% by 2030 (4). The associated healthcare costs, already estimated at $30 billion per year in the USA, are calculated to double in that period (5).

There are three categories of HF based on the measurement of left ventricular ejection fraction (LVEF). The most well-known and easiest to diagnose is heart failure with reduced ejection fraction (HFrEF, <40%). Heart failure with preserved ejection fraction (HFpEF, ≥50%) has different pathophysiological mechanisms (6) and is characterized by stiffening of the left ventricle and delayed early relaxation which contributes to elevated pressures in the left atrium (7). A newer category, heart failure with mid-range ejection fraction (HFmrEF, 40–49%), was established to encourage research into patients with different symptoms to the other two categories (8).

**Figure 1:**
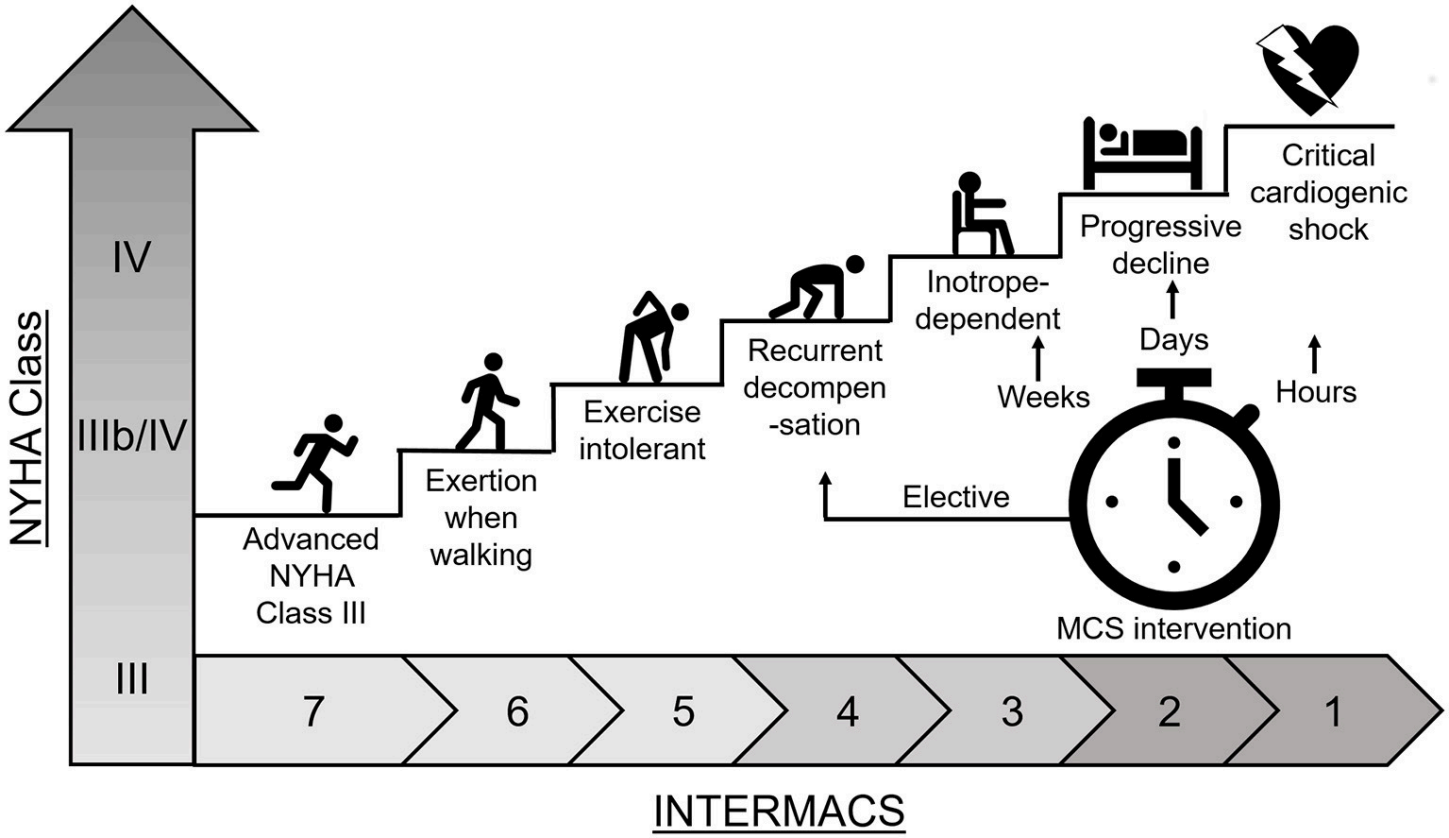
Heart failure disease progression. Heart failure disease progression and INTERMACS profile for determination of patient treatment with mechanical circulatory support (MCS).

The progression of HF has been classified into stages A-D by the American College of Cardiology (ACC) and into stages I-IV by the New York Heart Association (NYHA) (9, 10). The stages are interchangeable and are intended to reliably identify patients for the correct treatment. The Interagency Registry for Mechanically Assisted Circulatory Support (INTERMACS) profiling system comes in during stage C/NYHA IIIb (Figure 1) to determine whether the patient is suitable for transplant or mechanical circulatory support (MCS) which includes left ventricular assist devices (VADs) (11).

Heart transplantation remains the ultimate cure for HF with approximately 7000 hearts transplanted globally per year (12), yet a vast majority of patients are classed as ineligible for transplant (13) there is a great need for implantable mechanical circulatory support such as VADs. Despite VADs being life-saving therapy, adverse effects such as gastrointestinal bleeding, infection, stroke, and device thrombosis are still significant complications (14). As the inflammatory response can contribute to these and other complications, the aim here is to review the effect of VAD treatment on the inflammatory profile of HF patients, especially as it relates to leukocytes.

## Inflammation in heart failure

Heart failure is the result of stress to the heart due to a number of causative factors including coronary artery disease, hypertension, arrhythmias, diabetes, obesity, and age (8). The inflammatory profile of heart failure has been shown to differ depending on the ejection fraction category.

A common cause of HFrEF is myocardial infarction (2). MI-associated injury activates the innate immune system initiating a cascade of cytokines, chemokines, cell-adhesion molecules, and cell surface receptors designed to signal reparative cells to the area of injury (15). Activation of the immune system and release of inflammatory cytokines in the early stages helps maintain ventricular function (10) and is necessary to initiate repair of myocytes (15). During repair, the ventricle is remodeled which modifies size, shape, and contractility of the cardiac tissue cells (16, 17). The reduced blood flow at this time is compensated for by increasing blood volume through limiting salt release via urine. This places strain on the kidneys and the excess fluids can also affect the liver. Renal dysfunction causes derangements to metabolic and other circulating factors, which activates systemic inflammation (18). Excessive inflammation detrimentally affects repair of the heart and can culminate in cell damage and death leading to functional impairment of the myocardium through fibrosis and hypertrophy (15, 19, 20).

HFpEF on the other hand has a slightly different inflammatory profile. HFpEF can be caused by hypertension and has a bidirectional relationship with renal dysfunction (18, 21). These patients have increased blood levels of inflammatory cytokines such as tumor necrosis factor alpha (TNFα) interleukin-1 beta (IL-1β), and interleukin-6 (IL-6) (22) postulated to be linked to dysregulation of the cytokine network by regulatory T (Treg) cells (21, 23). IL-6 in particular is linked to myocardial fibrosis and increased myocardial stiffness (21).

The progression of HF can be measured through the heightened expression of inflammatory cytokines e.g., TNFα, IL-1, IL-6, IL-18, cardiotrophin-1, Fas ligand (FasL), monocyte chemoattractant protein 1 (MCP-1), IL-8, and macrophage inflammatory protein 1 alpha (MIP-1α) (21). These cytokines can be used as prognostic biomarkers (24–26) as circulating levels are directly proportional to deterioration in functional class and cardiac performance (27, 28). Increased levels of TNFα, IL-1β, IL-6, and galectin-3 (a galactose-specific lectin) levels can predict adverse outcomes (25). For example, galectin-3 is associated with inflammation and fibrosis, is a known mediator of heart failure development and progression (29) and is elevated significantly in NYHA I-IIIa vs. controls and further elevated in severe HF NYHA IIIB-IV (30). Another biomarker of HF includes N-Terminal-pro-BNP (NT-pro-BNP). Natriuretic peptides regulate volume and blood pressure homeostasis, but also have roles in the regulation of the immune response with receptors expressed on T cells, macrophages, and dendritic cells (31). Blood tests for these are available to diagnose congestive HF (32).

## Inflammation in response to ventricular assist devices

VAD implantation can alleviate symptoms whilst maintaining a reasonable quality of life for patients with end-stage heart failure either awaiting or ineligible for heart transplant (10). VADs can partially reverse some of the pathological processes involved in advanced HF and restore myocardial function (33). Inflammatory biomarkers are of diagnostic value to clinicians to classify HF and monitor progression, but this has not been translated routinely for use during VAD therapy to predict positive or negative outcomes.

Given the heightened inflammatory milieu of any potential VAD recipient, as discussed above, it is critical to better understand both the impact of the VAD on the patient's inflammatory profile and to address this during the development of new VADs. These same markers could also be used to monitor patients during VAD therapy for improvement or detecting and preventing adverse effects.

### Mechanisms through which VADs influence inflammation

The mechanisms through which VADs influence inflammation are yet to be fully understood. First generation VADs, such as the pneumatic VAD (PVAD; Thoratec, CA, USA), are pulsatile volume displacement pumps that mimic a physiological pulse but their size, durability, and complication rates has led to discontinuation of use. The second and third generation VADs are continuous flow pumps that have either an axial or centrifugal rotor. These pumps have the benefit of being smaller in size to ease implantation, but the continuous flow introduces high levels of non-physiological shear (34). All VAD types introduce two crucial elements that affect the immune system: high levels of shear stress and foreign materials with varying surface finishes.

Leukocytes are exposed repeatedly to the VAD at 1-min intervals (average blood flow at rest = 5 L/min) (35). Brief exposure to high shear sensitizes platelets to activation under subsequent low insult shear, so it is reasonable to presume leukocytes might be affected the same way (36). It has been suggested that shear stress activates leukocytes through structural changes rather than ligand-induced signal transduction (37). Interestingly, repeated mechanical deformation at 25 dynes/cm^2^ of primed neutrophils can return them to a resting state (38), but above this leads to structural disruption (39).

Leukocytes also contend with different biomaterials and varying surface finishes throughout the VAD. The surface properties of these biomaterials drive the foreign body response. This response begins with protein adsorption (i.e., albumin, fibrinogen, globulins) at the biomaterial surface creating a highly dynamic matrix which can make the surface inert, or encourage cellular activation and adhesion (40). Surface roughness is an important factor to consider as long-term continuous-flow VADs (CF-VADs) have highly polished, smooth surfaces near the rotating impeller (41) to reduce adhesion of proteins and cells (41). Some VADs, both continuous and axial flow, have sintered titanium microsphere surfaces to encourage cellular and protein adherence to form a stable, densely adherent biological lining (42). This biological lining is hypothesized to “hide” the foreign material and reduce the immune response.

**Figure 2:**
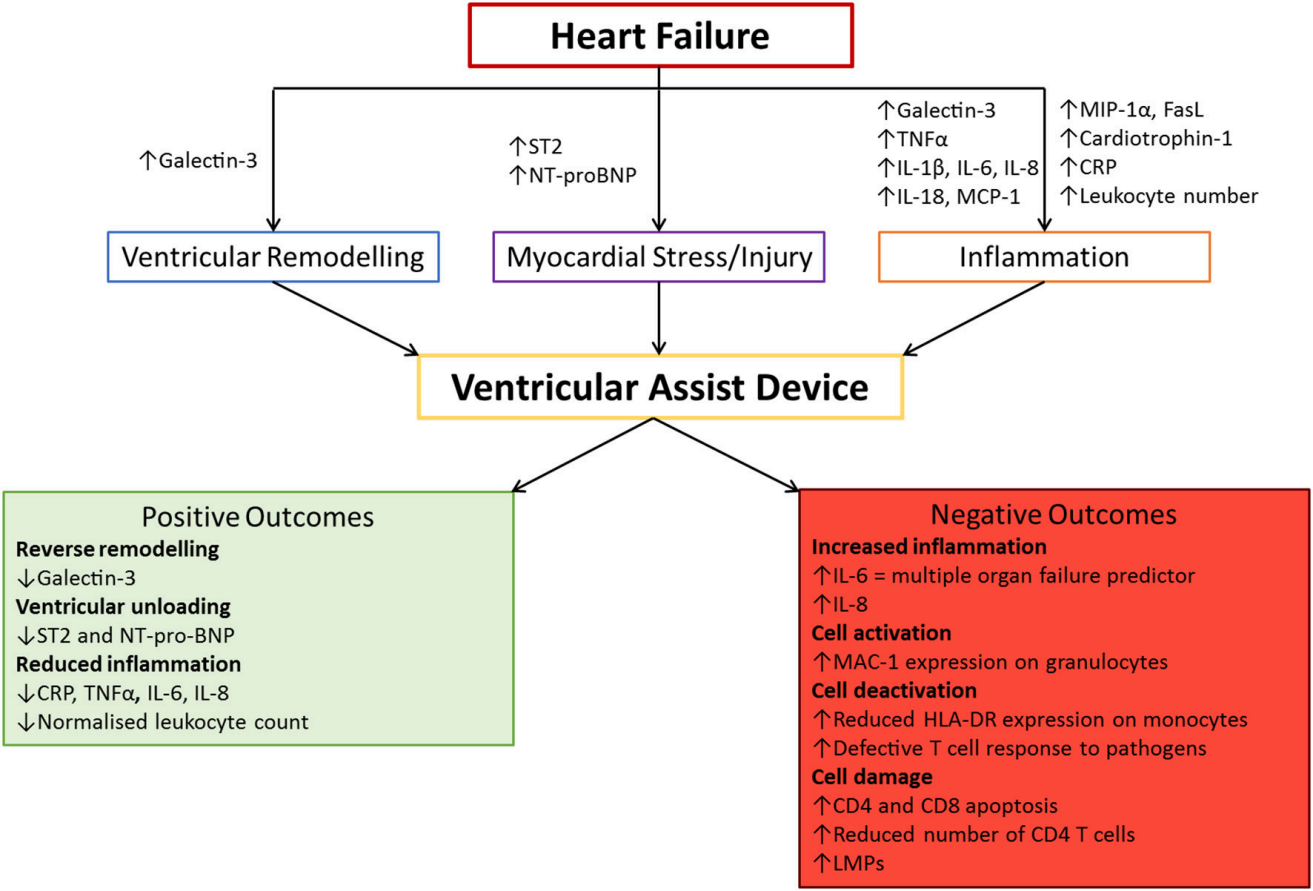
The association between VAD implantation and inflammation. Heart failure has its own distinct inflammatory profile. Ventricular assist device (VAD) implantation can have both positive and negative outcomes which impact the efficacy of using this treatment. These markers can be used to monitor the progression of VAD therapy and predict adverse events for early intervention purposes. CRP, C-reactive protein; IL, Interleukin; MCP-1, Monocyte Chemoattractant Protein 1; MIP-1α, Macrophage Inflammatory Protein 1 alpha; NT-proBNP, N-Terminal pro Brain Natriuretic Peptide; TNFα, Tumor Necrosis Factor alpha.

The complex interactions between leukocytes and VADs, combined with the pre-existing inflammatory profile of the VAD recipient, can lead to a plethora of possible inflammatory outcomes (Figure 2). This could make their use as biomarkers difficult, but there are some inflammatory mediators that indicate the effectiveness of VAD therapy. The following section has been summarized in Table 1.

**Table 1:** The effect of VAD implantation on inflammatory parameters.

**Study**	**Effect of VAD implantation**
**INFLAMMATORY AND CARDIAC BIOMARKERS**	
Ahmad et al. (29) Yu et al. (36)	↓CRP halved compared to pre-operative values within 60 days ↓CRP levels in pediatric VAD patients within 1 month
Ahmad et al. (29)	↓NT-proBNP >60 days post-implantation but still abnormally high
Ahmad et al. (29) Caselli et al. (37)	↓ST2 decreased in CF-VAD patients but still abnormally high ↓ST2 normalized within 1 month in CF-VAD patients
Ahmad et al. (29) Coromilas et al. (38) Lok et al. (38)	↓Galectin-3 decreases with VAD placement ↑Galectin-3 becomes elevated, exceeding pre-operative levels after 6 months of VAD therapy
**CYTOKINES**	
Bedi et al. (41)	↓TNFα decreased slightly in myocardium post-VAD but still elevated compared to controls
Corry et al. (44) Bedi et al. (41) Caruso et al. (39) Loebe et al. (47)	↓IL-6 initially elevated but declines below pre-operative levels after 6 weeks ↓IL-6 decreased in the myocardium following VAD support ↑IL-6 increase during VAD support indicative of multiple organ failure ↑IL-6 blood levels higher in CF-VADs than PF-VADs
Corry et al. (44) Grosman-Rimon et al. (30)	↓IL-8 initially elevated but declines below pre-operative levels after 6 weeks ↑IL-8 blood levels increase to greater than pre-operative levels in CF-VAD by 9 months
**LEUKOCYTES**	
Woolley et al. (49)	↓Decline in peripheral blood leukocyte count with VAD implantation after 60 days ↑Increase in MAC-1 expression on granulocytes with HMII until POD 120
Kirsch et al. (57)	↓Reduced expression of HLA-DR on monocytes in VAD recipients
Ankersmit et al. (52, 53) Kimballet al. (54, 55)	↓Decrease in CD4 T cells and higher levels of CD4 and CD8 T cell apoptosis ↓Defective T cell response to inflammatory stimuli
**MICROPARTICLES**	
Diehl et al. (61) Sansone et al. (62) & Diehl et al. (61)	↑Increased levels of LMPs in VAD patients ↑Increased levels of LMPs in both HMII and HVAD

*Papers describing inflammatory effects by VADs characterized into inflammatory and cardiac biomarkers, cytokines, leukocytes, and microparticles*.

### Clinical studies of the inflammatory effects of VADs

Clinical studies that measure changes in circulating mediators have provided insight into how effective VAD therapy is. Biomarkers most often used include C-reactive protein (CRP), a common biomarker of systemic inflammation (43), N-terminal brain natriuretic peptide (NT-proBNP) used to establish prognosis in heart failure (44), suppressor of tumorigenicity 2 (ST2), a member of the IL-1 receptor family, that signposts cardiac remodeling and tissue fibrosis (45), and galectin-3, which is associated with higher risk of mortality (46). Inflammatory cytokines, mainly IL-6, TNFα, and IL-8, have also been studied in patients implanted with VADs and provide insight into cardiac function (21, 25).

#### Inflammatory and cardiac biomarkers

There is an initial elevation of inflammatory markers in response to VADs but this is largely due to the surgical procedure itself. Increased CRP levels are consistent with inflammation and a further increase is normal in response to the trauma of surgery (47). End-stage HF patients already have around 8-fold higher levels of circulating CRP than the typical healthy reference value of 0–5 mg/L in serum (48). VAD therapy improves the inflammatory profile of the patient with levels of CRP halved compared to pre-operative values within 60 days after implantation (33). A similar profile is seen in pediatric patients; pre-VAD CRP levels are abnormally high due to HF (~35 mg/L), are exacerbated by surgery, and begin to decrease 1 month post-implantation with levels returning to the healthy range after 5 months (49).

Circulating NT-proBNP levels decrease significantly in VAD patients between 24 h pre-implantation and >60 days post-implantation but still remain abnormally high (33). This decrease is indicative of normalizing blood volume and pressure which lessens the strain on the kidneys. However, the severity of kidney damage and its bidirectional relationship with HFpEF could be the reason NT-proBNP levels remain abnormal.

ST2 isconsidered to be a superior cardiac stress biomarker as levels are not affected by age, BMI, history of HF, anemia, or renal failure (45). Levels of ST2 decrease in patients implanted with continuous-flow VADs, but remain abnormally high >60 days post-implantation (33). This suggests cardiac improvement but not to a point where the patient is no longer at high risk of mortality. In contrast, those implanted with axial-flow VADs showed that elevated pre-operative ST2 levels were normalized 1 month post-VAD (50) suggestive of cardiac de-stress and repair. This would indicate that the axial-flow design is more favorable toward reverse remodeling.

Galectin-3 levels show a similar trend with higher levels pre-operatively in HF patients compared to healthy controls (51) and a decrease during VAD placement (33). However, galectin-3 levels become elevated again and can even exceed pre-operative levels after 6 months VAD therapy (30, 51). The reasons for this are unknown but it might be that the VAD is not supporting myocardial recovery and that galectin-3 levels identify patients at higher risk of death.

#### Cytokines

Circulating cytokines, especially TNFα, IL-6, and IL-8 that regulate systemic inflammation, are also of interest in HF and VADs. Elevation of these is linked to poor outcomes (52). TNFα is elevated in severe HF and can predict mortality (53). Decreases of TNFα within the myocardial tissue post-VAD implantation indicate a tissure repair response to VAD treatment although levels remain higher than in controls(54–56).

IL-6 is increased immediately after VAD implantation with a decline to below pre-operative levels after 6 weeks (57). However, patients with higher pre-operative IL-6 (such as those with HFpEF) are more susceptible to poor early outcomes (52). An increase in IL-6 post-implantation is associated with multi-organ failure (58), the main cause of death during early phase MCS (59). Blood IL-6 concentrations also differ by VAD type being higher with CF-VAD than with pulsatile (PF-VAD) flow devices (60). This is likely related to the exposure of leukocytes to non-physiological flow and high shear levels in CF-VADs leading to increased activation. As for TNFα, there isan overall reduction in IL-6 in the myocardium following VAD support (54).

IL-8 showssimilar patterns of change with an immediate increase post-implantation and a decrease to pre-operative levels by 4–6 weeks post-operatively (52, 57). There are also differences by VAD type; while pulsatile and continuous VADs both show a post-operative decline in IL-8 (61), levels had increased to greater than pre-operative levels by 9 months post-implantation with continuous flow devices (43). Again, this could be a feature of the shear stress effects on leukocyte activation.

#### Leukocytes

Changes in circulating leukocyte numbers and phenotype can also provide more detail about the inflammatory changes associated with VAD implantation. Pre-operatively, VAD patients have leukocyte counts at the higher end of normal range, consistent with their inflammatory profile. Overall, VAD implantation seems to be associated with a decline in peripheral blood leukocyte counts (62, 63). In patients implanted with either a PVAD, HeartMate II (HMII; Thoratec), or HVAD (HeartWare, MA, USA), these leukocyte counts initially increased significantly (post-operative day (POD) 14) then decreased significantly to below pre-op levels by POD 60 in all VAD types (62). This would suggest an improvement in the inflammatory profile.

Despite normalization of leukocyte counts, cell phenotype is changed ina VAD-dependent manner. Expression of the activation marker, macrophage antigen-1 (MAC-1), on granulocytes is a marker for systemic inflammation (64). MAC-1 was increased significantly in HMII patients at POD 14 and did not return to pre-operative levels until POD 120. For HVAD patients, an initial small increase in MAC-1 expression at POD 14 was seen with a return to pre-operative levels by POD 60. This has been linked to the higher rates of and susceptibility to infection in HMII patients compared to HVAD patients (62) likely due to design differences: HMII being an axial-flow pump and the HVAD being a centrifugal pump. Variable increase in MAC-1 expression might identify patients vulnerable to recurrent infection and inform intervention.

Additionally, there are wider immune system alterations caused by VADs. Patients implanted with pulsatile flow VADs have a selective reduction in CD4^+^ T cells, defective proliferative responses to stimuli such as staphylococcal enterotoxin B, and higher levels of apoptosis in CD4^+^ and CD8^+^ T cells in comparison to medically treated NYHA class IV patients (65–68). Disruption of adaptive immune cells contribute to risk of recurrent infection and sepsis. The effects of continuous-flow devices on lymphocytes are suspected to be less severe, although the data are limited. The one study we could identify showed increased apoptotic activity in CD4^+^ T cells up to 4 weeks post-operatively which normalized to baseline after 7 weeks (69).

Potential deactivation of monocytes through a reduced expression of HLA-DR has also been observed in VAD recipients and might be a marker of mortality. Patients in intensive care who died within the first 30 days of implantation exhibited a lower percentage of monocytes expressing HLA-DR than those who survived (70). This might be due to immunoparalysis which during the immediate support phase could hamper tissue repair for end-organ recovery (52).

#### Microparticles

Recent years have seen a burgeoning interest in the use of microparticles (MPs) as biomarkers of inflammatory diseases such as psoriasis, preeclampsia, pulmonary hypertension, and heart failure (71, 72). MPs are cell-derived vesicles between 0.1 and 1 μm in size that are formed from the outward blebbing of the plasma membrane due to cell stress (71). High levels of artificial mechanical stress produced by the VAD disrupts cells leading to damage, apoptosis, and stress-induced MP formation (72). MPs can be derived from platelets (PMPs), leukocytes (LMPs), and endothelial cells (EMPs) and have strong inflammatory and pro-thrombotic properties. Significantly elevated levels in VAD patients could be predictive of adverse events (73). Increased levels of LMPs have been demonstrated in VAD patients compared to historical controls and were elevated independently of leukocyte counts, suggesting that LMPs might be a marker of vascular inflammation (74). VAD type might not affect this parameter as both HMII and HVAD patients had increased LMPs (74, 75).

#### Summary

Overall, VADs can improve the inflammatory profile of HF patients through reducing high leukocyte counts and pro-inflammatory cytokine levels. VAD design, such as biomaterial choice and levels of shear stress, greatly affects whether inflammation and the immune system are affected positively or negatively.

Positive effects identified here includes improving pre-operative levels of inflammatory markers and cell phenotypes [e.g., quicker reduction in granulocyte activation in HVAD patients than HMII (62)]. Negative effects identified here includes monocyte deactivation, T cell apoptosis (69, 70), and higher inflammatory cytokine levels (43, 60). Future studies with newer devices of very different design to the HMII such as the HVAD and Heartmate III (HMIII) might reveal an improvement in how these VADs interact with the inflammatory response and the immune system.

### Preclinical studies for VAD development

As discussed above, both heart failure and VAD implantation can affect the inflammatory profile of the patient. Despite this, the initial stage of VAD development focuses mainly on the effects on red blood cells through analysis of haemolysis (76). The need for total blood damage evaluation is not yet widely recognized and the effects of new VAD designs on leukocyte number and function and the inflammatory response is only slowly being included in preclinical evaluation. Studying the impact of VADs on the inflammatory response during preclinical testing would allow better understanding and improvement of the device.

However, there are no published studies of the inflammatory markers described above during *in vitro* VAD testing although, such approaches are being incorporated into preclinical studies in animals. In a study that involved implanting nine dogs with and six dogs without VADs, TNFα levels in blood and renal tissues did not differ after 6 h (77). Changes in leukocyte counts and activation profiles have been explored in animal preclinical studies. Cows implanted with the EVAHeart or HeartMate II had increased in monocyte tissue factor, monocyte-platelet and granulocyte-platelet aggregates in the first few days post-operatively. While levels decreased over the next 30 days they remained higher than pre-operative levels and are suggestive of increased risk of thrombosis (78). However, when the HVAD was implanted into sheep, leukocyte count remained within normal ranges (79). This shows that the impact of very different VAD designs on inflammation can be detected *in vivo*.

There are limitations to what can be measured during *in vitro* testing due to the models typically being simple blood only loops but the effects on leukocytes can be assessed. To this end, our own *in vitro* studies showed an increase in leukocyte-derived microparticles emerging in bovine and ovine blood circulated through the CentriMag™ (63, 80). This increase is important as the CentriMag is considered the ‘gold standard' for blood handling yet there is clearly a so far unappreciated shear-effect on leukocytes.

## Summary

Heart failure is characterized by an inflammatory response, initiated by injury during myocardial infarction or the repetitive stress of hypertension on cardiac cells (15, 21, 22). Acute inflammation initially helps maintain ventricular function and support tissue repair but eventually this remodels the ventricle leading to altered function (16, 17). Chronic inflammation can lead to myocardium dysfunction due to fibrosis, hypertrophy, and cell death (15). Consequently, pro-inflammatory mediator expression can be used to map progression of HF, is proportional to functional class (21, 24–28), and can predict adverse outcomes (25). Interestingly, right ventricular failure (RVF) post-left VAD implantation is an emerging major complication. Post-operative RVF has been associated with higher pre-operative leukocyte counts and CRP levels suggesting that systemic inflammation may be contributory after LVAD implantation (81).

VADs have the potential to partially reverse the pathological processes involved in the progression of HF and restore myocardial function (33). The effectiveness of this can be monitored as a decline in biomarkers such as NT-proBNP, ST2 and the cytokines IL-6 and IL-8 although there are differences in the pattern of response for pulsatile- vs. continuous-flow devices (60, 61, 82). Irrespective of the type of device implanted, the inflammatory profile of patients pre-VAD can be linked to poor early outcomes (52).

Recent advances in VADs have led to the development of smaller pumps with better blood damage profiles compared to their predecessors. The differential effects of continuous-flow vs. pulsatile flow devices have been noted and there is a growing trend toward developing pumps with pulsatile capability, such as the HeartMate III (HMIII, Thoratec), to better maintain the natural rhythm of the body and reduce blood damage (83). VAD research is improving our knowledge of the effects of novel biomaterials and shear stress on blood. Yet, mostly for practical and ethical reasons, preclinical studies use healthy human or large animal blood (bovine/ovine/porcine) instead of blood from heart failure patients which has an inherently different inflammatory phenotype.

Evidence that VAD treatment improves these inflammatory parameters and elucidation of the associated mechanisms could benefit the design of future heart pumps for improved longevity and quality of life for patients. These changes in inflammatory markers alone could also be beneficial to clinicians in monitoring VAD therapy and early-intervention for adverse events such as sepsis.

## Author contributions

GR and IP: conception and design of the review, drafting of the manuscript, and critical revision of the manuscript. SA: drafting of the manuscript, critical revision of the manuscript. FB: critical revision of the manuscript. CT: conception and design of the review, drafting of the manuscript, critical revision of the manuscript.

### Conflict of interest statement

GR and SA are employees of Calon Cardio – Technology Ltd (Calon). IP is employed by Scandinavian Real Heart AB. The remaining authors declare that the research was conducted in the absence of any commercial or financial relationships that could be construed as a potential conflict of interest.

## Abbreviations

ACC: American College of Cardiology
CF: Continuous Flow
CRP: C-Reactive Protein
EMP: Endothelial-derived Microparticle
HF: Heart Failure
HFmrEF: Heart Failure with mid-range Ejection Fraction
HFpEF: Heart Failure with preserved Ejection Fraction
HFrEF: Heart Failure with reduced Ejection Fraction
HLA-DR: Human Leukocyte Antigen-D Related
HMII: HeartMate 2
HVAD: HeartWare Ventricular Assist Device
IL: Interleukin
INTERMACS: Interagency Registry for Mechanically Assisted Circulatory Support
LMP: Leukocyte-derived Microparticle
LVEF: Left Ventricular Ejection Fraction
MCP-1: Monocyte Chemotactic Protein 1
MCS: Mechanical Circulatory Support
MIP-1α: Macrophage Inflammatory Protein 1 alpha
MP: Microparticle
NT-proBNP: N-Terminal pro Brain Natriuretic Peptide
NYHA: New York Heart Association
PF: Pulsatile Flow
PMP: Platelet-derived Microparticle
POD: Post-Operative Day
PVAD: Pulsatile Ventricular Assist Device
TNFα: Tumor Necrosis Factor alpha
Treg: egulatory T cells
VAD: Ventricular Assist Device.
